# Pharmacological effects and application prospects of mussel adhesive proteins

**DOI:** 10.3389/fphar.2025.1704475

**Published:** 2025-12-09

**Authors:** Junsha An, Zengmiao Hou, Jiaqi Li, Qian Bi, Shile Huang, Cheng Peng, Fu Peng

**Affiliations:** 1 West China School of Pharmacy, Sichuan University, Chengdu, China; 2 State Key Laboratory of Southwestern Chinese Medicine Resources, Chengdu University of Traditional Chinese Medicine, Chengdu, China; 3 Xi’an DeNovo Hith Medical Technology Co., Ltd., Xi’an, China; 4 Department of Radiation Oncology, Peking Union Medical College Hospital, Chinese Academy of Medical Sciences and Peking Union Medical College, Beijing, China; 5 Key Laboratory of Drug-Targeting and Drug Delivery System of the Education Ministry and Sichuan Province, Sichuan Engineering Laboratory for Plant-Sourced Drug and Sichuan Research Center for Drug Precision Industrial Technology, Sichuan University, Chengdu, China

**Keywords:** mussel adhesive proteins, adhesion, biomedical applications, drug delivery system, tissue engineering

## Abstract

Mussel adhesive proteins (MAPs) are natural proteins derived from *Mytilus edulis*, renowned for their exceptional adhesive properties. These proteins, rich in 3,4-dihydroxyphenylalanine (DOPA) residues, enable mussels to adhere firmly to surfaces in challenging environments. Due to these unique biochemical and mechanical characteristics, MAPs have attracted significant attention in the biomedical field, offering promising applications in wound healing, drug delivery, tissue engineering, and cosmetics. Recombinant MAPs (rMAPs), in particular, hold great potential due to their enhanced properties, including antioxidant, anti-inflammatory, antibacterial, and cell-protective effects. They are increasingly being explored for their role in tissue repair, skin regeneration, and targeted drug delivery systems. Despite challenges in recombinant production, toxicity control, and underwater adhesion efficiency, ongoing advancements in genetic engineering and protein design are expanding the application prospects of rMAPs. This review explores the structure, pharmacological effects, and biomedical applications of MAPs, with a focus on the potential of rMAPs in precision medicine, drug delivery, and tissue regeneration, while highlighting the challenges and future directions for their development.

## 1 Introduction

Mussel adhesive proteins (MAPs) are a class of natural proteins with unique adhesive properties, widely found in *Mytilus edulis*. Their most prominent feature is the ability to firmly adhere to various surfaces in moist, salt-rich environments. This ability arises from the presence of 3,4-dihydroxyphenylalanine (DOPA) residues in their molecular structure, allowing MAPs to generate strong adhesive forces through multiple intermolecular interactions, such as oxidation crosslinking, charge interactions, metal-phenolic coordination, hydrogen bonding, and hydrophobic interactions (Waite, 2017; Liang et al., 2018). During mussels’ physiological processes, the adhesive proteins form gel-like aggregates in their foot via a special secretion mechanism, ensuring the protein’s adsorption and extension on surfaces under extreme conditions (such as low pH, low ionic strength, and high reducing environments), eventually solidifying into a robust adhesive layer (Yang B. et al., 2018). This characteristic is crucial not only for mussels’ ecological adaptation but also provides significant potential for biomedical applications.

It is interesting to notice that emerging natrual products exert various pharmacological effects, including anti-inflammation, antioxidation, cardioprotection, neuroprotection and anticancer effect (Shi et al., 2020; Li et al., 2021; Zou et al., 2021; Liu and Liu, 2018; Wang Y. et al., 2024). In recent decades, the unique biochemical and mechanical properties of MAPs have garnered extensive attention prospects in the biomedical field. Their ability to promote wound healing and tissue regeneration, as well as their potential use in drug delivery systems, tissue engineering, and immunotherapy, has gained increasing recognition (Guo et al., 2020; Quan et al., 2019). The bioactive properties of MAPs extend beyond their adhesive capabilities. Studies have shown that MAPs possess antioxidant, anti-inflammatory, antibacterial, and cell-protective properties, making them promising candidate materials for biomedical applications (Zhao et al., 2023). Furthermore, the biocompatibility and biodegradability of MAPs enhance their appeal as a safe and sustainable medical material (Zhang et al., 2020; Madhurakkat Perikamana et al., 2015).

In contrast to previous reviews that mainly focused on the structural chemistry and adhesion mechanisms of MAPs, this review aims to explore the basic structure and pharmacological characteristics of MAPs, with a focus on their current research applications in wound healing, drug delivery, tissue engineering, and cosmetics and skincare products. Furthermore, we systematically compare recombinant MAPs (rMAPs) with other natural biomaterials and catechol-based synthetic systems, highlighting their distinctive advantages and limitations in translational applications, providing theoretical support for future application studies.

## 2 Fundamental structure and modifications of MAPs

The unique molecular structure and amino acid sequence features of MAPs allow them to adhere to various surfaces underwater, adapting to tidal changes and the impact of waves. MAPs have a complex structure with high functionality, especially certain structural domains that are critical to their adhesive capabilities.

### 2.1 Molecular architecture of MAPs

The basic structural unit of MAPs consists mainly of multiple amino acid sequences with various post-translational modifications. Regions rich in tyrosine and glycine are considered to be the key functional areas of MAPs. Glycine, the smallest amino acid, often forms flexible connecting regions in the sequence of MAPs, which helps the protein adapt to changes in complex environments (Ou et al., 2020). Tyrosine, on the other hand, undergoes oxidation to form DOPA, playing a core role in the adhesion process. The high content of DOPA is a distinctive feature that sets MAPs apart from other adhesive proteins (Deepankumar et al., 2022).

In the structure of MAPs, several key structural domains play crucial roles. Particularly, the linker domain and the adhesive domain are responsible for the stability of the protein and its adhesion to the surface (Zeng et al., 2018). The linker domain is usually composed of sequences rich in glycine and proline, which can form helical or folded structural units. These amino acids improve the spatial stability of the protein and enhance its adhesive function by interacting with other proteins or surfaces, making the adhesion more durable. The adhesive domain, especially regions rich in tyrosine and DOPA, directly participate in the underwater adhesion process (Yoon et al., 2025; Shin et al., 2022). DOPA contains a phenolic hydroxyl group, a structure that allows it to form stable complexes with metal ions (such as iron and copper) and other molecules. It can also interact with surrounding molecules or surfaces through hydrogen bonding, thus enhancing adhesion (Kim et al., 2018).

**FIGURE 1:**
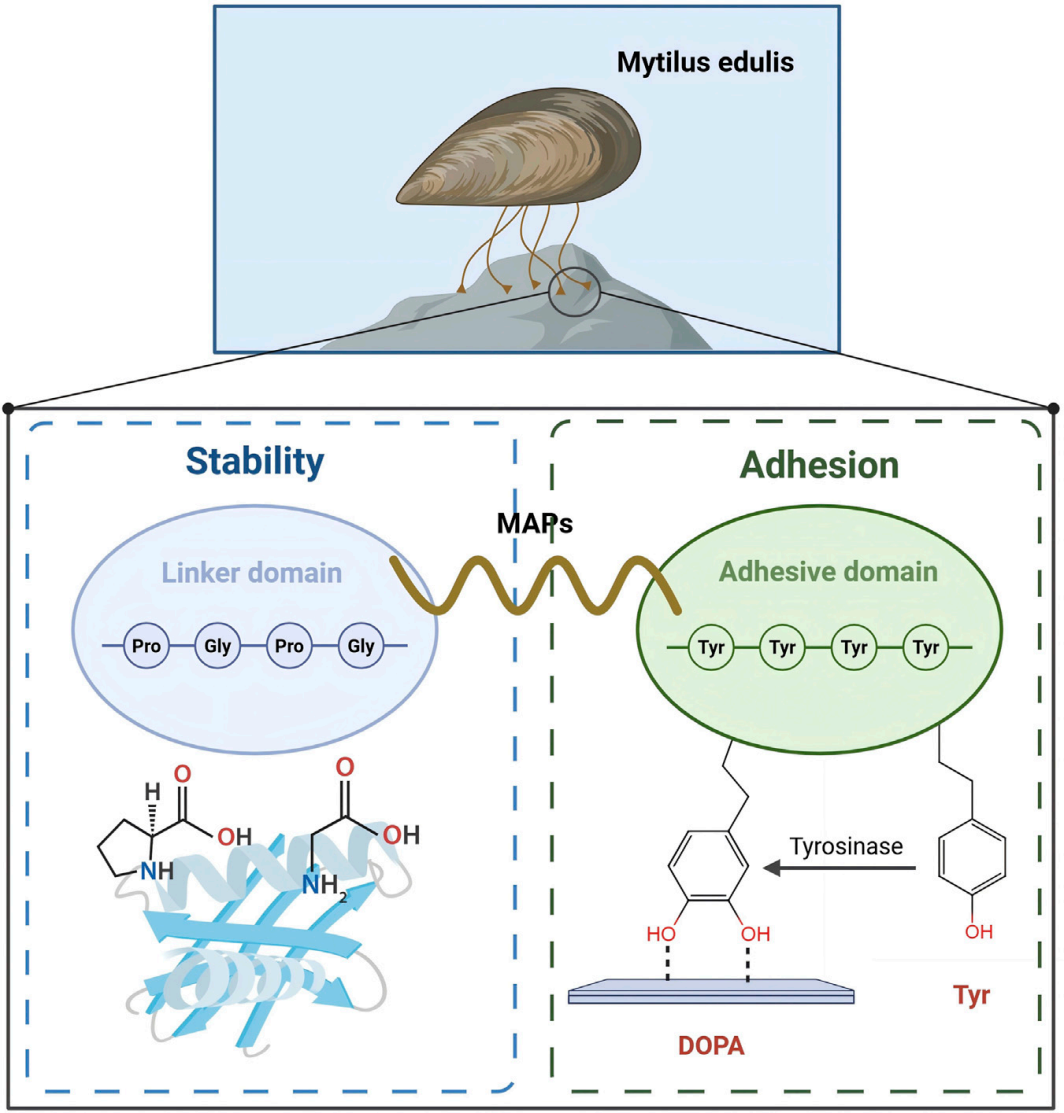
Structure and functional regions of MAPs.

In summary, the structural characteristics of MAPs, such as the regions rich in tyrosine, glycine, and DOPA, provide them with powerful adhesive properties in underwater environments (Figure 1). These structural units and functional domains work together through multiple mechanisms, including chemical bonds, metal ion coordination, hydrogen bonding, and hydrophobic interactions, ensuring that mussels can firmly attach to various surfaces. Through in-depth research on these adhesive properties and structural domains, we can not only better understand the biological adhesion mechanisms in nature but also provide theoretical and technical support for developing new types of biological adhesives and other high-performance materials.

### 2.2 Impact of structural modifications on adhesive properties

Traditional adhesives often lose their adhesive power due to the influence of water, whereas MAPs can maintain stable adhesion in water. Research has shown that the thin film formed by Mefp-1 (a type of MAPs) on a platinum surface exhibits good adhesive properties, and the formation and compactness of this film can be controlled under different potentials. Mefp-1 adsorbs to surfaces through both electrostatic and non-electrostatic interactions, effectively blocking electroactive sites, which allows it to retain strong adhesive strength in underwater environments (Zhang et al., 2017).

The structural modification of MAPs mainly involves improving their adhesion properties in aquatic environments by mimicking the adhesion mechanism of mussels. For example, CMC-DA hydrogels, which are carboxymethyl cellulose (CMC) molecules conjugated with dopamine (DA), have been developed. These hydrogels undergo enzyme cross-linking under the catalysis of horseradish peroxidase (HRP) and hydrogen peroxide (H_2_O_2_), significantly enhancing their adhesion strength (Zhong et al., 2019).

Moreover, the co-precipitation effect of MAPs provides them with broader adaptability. Studies have altered the ionic properties and pH conditions of polymers, creating non-charged, biodegradable polyester materials that can quickly form co-precipitates in water, exhibiting excellent underwater adhesive properties across a wide range of pH and ionic strengths (Narayanan et al., 2020). This design not only overcomes the sensitivity of traditional co-precipitation adhesives to changes in pH and salinity but also enhances their potential for application in complex environments.

MAPs are inherently insoluble in water. Through recombinant DNA technology, Pilakka Veedu A et al. successfully developed a mussel adhesive protein fusion protein that maintains high solubility and stability in aqueous solutions (Pilakka et al., 2023). For example, Foot Protein 1 (Fp1), a type of MAPs, when fused with the highly water-soluble ice-nucleation protein K (InaKC), results in a fusion protein that exhibits lower adhesive strength but better water solubility and stability. Once separated by protease cleavage sites, the fusion protein can regain its adhesive properties. This demonstrates that by altering the structure and fusing other proteins, the water solubility and adhesive properties of MAPs can be flexibly controlled to meet different application needs.

MAPs can also promote cell adhesion through interactions with cell surface receptors, a characteristic crucial for the design of tissue engineering and biomedical materials. By fusing MAPs with functional peptides derived from laminin, fusion proteins have been designed that significantly improve adhesion to A549 cells, further proving the potential of MAPs in cellular biomaterials (Wang et al., 2017).

Additionally, studies show that the secretion of MAPs is time-regulated, allowing their adhesive abilities to adjust according to environmental conditions at different time points. This regulatory mechanism optimizes the surface interactions of MAPs and facilitates the assembly of foot proteins (Petrone et al., 2015).

In conclusion, MAPs exhibit excellent performance in underwater adhesion and cell adhesion, among other fields. Their unique physicochemical properties, such as time-regulated secretion mechanisms and adjustable water solubility and adhesiveness, enable them to function effectively in various environments. These characteristics offer vast prospects for the application of MAPs, especially in replacing traditional adhesives and developing new biomaterials, making them of significant importance.

### 2.3 Comparative composition of MAPs across species and secretion conditions

MAPs constitute a heterogeneous family rather than a single molecule. Their amino-acid composition, sequence motifs, and post-translational modifications (PTMs) differ markedly among species and are further modulated by secretion and curing conditions (DeMartini et al., 2017). Comparative analyses across Mytilus, Perna, and Bathymodiolus species reveal systematic variations reflecting habitat and functional specialization (Rees et al., 2019). Intertidal Mytilus species exhibit high tyrosine and lysine contents, with efficient enzymatic conversion of tyrosine to DOPA, producing strong wet adhesion through multivalent catechol–metal coordination. In contrast, tropical or deep-sea mussels synthesize proteins richer in glycine, proline, and cysteine, imparting chain flexibility, coacervate formation, and redox buffering capacity. Consequently, DOPA/Lys-rich proteins enhance tissue binding and drug retention, low-complexity Gly/Pro-rich domains promote slow release, and Cys-rich variants improve oxidative stability and compatibility with sensitive biomolecules (Li and Zeng, 2016). MAPs also differ in motif architecture: DOPA-rich decapeptide repeats and Lys–Tyr–Gly (KYG) clusters concentrate catechol groups for adhesion; His-rich segments enable pH- or metal-responsive binding; and Cys motifs form disulfide networks for structural stabilization. These sequence features are fine-tuned by PTMs such as DOPA hydroxylation, phosphorylation, glycosylation, and controlled oxidation, which together regulate charge, hydrophilicity, and redox balance.

The secretion of MAPs during byssus formation is dynamically regulated by environmental cues (Priemel et al., 2020). Secretion begins in an acidic, reducing milieu that preserves DOPA in its catechol form, favoring surface adsorption, followed by exposure to oxygen and Fe^3+^ that drives oxidative curing and covalent cross-linking. This sequential release of adhesive, cohesive, and protective proteins produces a gradient from compliant interface to rigid plaque core (Valois et al., 2020). Variations in pH, redox potential, or metal ion concentration between species or experimental conditions thus alter the molecular mixture and ultimately affect mechanical and biological performance. The same protein framework can therefore yield distinct materials depending on environmental modulation, emphasizing the adaptive nature of MAP chemistry.

These compositional and environmental factors collectively shape pharmacological behavior. High DOPA/Lys content confers prolonged adhesion and residence at target tissues, low-complexity sequences support coacervate-based diffusion control, and Cys- or redox-active domains mitigate oxidative stress and inflammation. MAP systems enriched in catechol–metal motifs can further act as antimicrobial or stimuli-responsive coatings (Santonocito et al., 2019). Pharmacological efficacy thus arises not from a single residue but from the combinatorial interplay of motifs and PTMs regulated by secretion context. Recognizing this interspecies and condition-dependent diversity provides a mechanistic foundation for designing MAP-inspired biomaterials with tunable adhesion, controlled release, and improved biocompatibility.

### 2.4 Expansion of the MAP family: newly identified components and structural diversity

Recent transcriptomic and proteomic investigations have substantially broadened the MAP family beyond the classical Mfp-1–Mfp-6 set.

In *Mytilus californianus* foot glands, approximately fifteen previously unrecognized candidates (provisionally named Mcfp-7p to Mcfp-19p) were identified. These proteins share hallmark MAP features—including signal peptides, basic isoelectric points, and Lys/Gly/Tyr-rich compositions. Among them, Mcfp-7p and Mcfp-8p are short KYG (Lys–Tyr–Gly) repeat peptides, while Mcfp-10p–Mcfp-12p are longer sequences enriched in His/Gly or Cys residues. Their strong expression in both the phenol and accessory glands suggests roles in the early stages of plaque formation (DeMartini et al., 2017). In addition, preCOL-P, preCOL-D, and preCOL-NG are the main components of the inner core fibrils of the mussel foot filaments, and they are modified with glycine. Furthermore, there are the foot-specific filamentous matrix proteins PTMP (proximal filamentous matrix protein) and DTMP (distal filamentous matrix protein) (Xu S. et al., 2024).

The expanded catalog of MAPs fundamentally reshapes our understanding of how molecular diversity governs pharmacological performance and materials design. Each subclass provides distinct chemical functionalities that map to specific therapeutic outcomes: DOPA and Lys-rich proteins enhance surface adhesion and local residence; cysteine- and redox-regulating proteins protect biomolecules from oxidative stress. Together, these discoveries reveal that pharmacological efficacy arises from the synergistic interplay of multiple motifs rather than a single adhesive residue.

## 3 Pharmacological effects of MAPs

In recent years, MAPs have received widespread attention for their pharmacological effects in areas such as anti-inflammatory, antibacterial, wound healing, and tumor immunotherapy. Researchers have found that MAPs can improve the process of skin tissue repair, reduce inflammation, and promote wound healing, offering new perspectives for clinical treatment.

### 3.1 Anti-inflammatory effects and prevention of hyperpigmentation

MAPs have shown anti-inflammatory effects in the application of skin inflammation and wound healing. Studies have shown that rMAPs r-fp-151-V can effectively inhibit the expression of inducible nitric oxide synthase (iNOS) and cyclooxygenase-2 (COX-2) in mouse macrophages stimulated by lipopolysaccharide (LPS), thereby reducing the production of nitric oxide (NO). Furthermore, when applied to keratinocytes after ultraviolet (UV) exposure, r-fp-151-VT significantly reduced the expression of iNOS and COX-2 and decreased the production of inflammatory cytokines such as IL-1β, IL-6, and TNF-α. This result indicates that MAPs have particularly significant anti-inflammatory effects on skin cells, especially in the treatment of UV-induced skin inflammation, where it demonstrates strong efficacy (Ahn et al., 2019).

MAPs have also shown potential in preventing and treating hyperpigmentation. Post-inflammatory hyperpigmentation (PIH) is a common complication after skin inflammation, especially more pronounced in individuals with darker skin tones. Research has found that MAPs can significantly reduce the likelihood of PIH occurring when used as a wound dressing. MAPs were found to be equally effective in reducing pigmentation compared to the potent steroid drug fluticasone propionate. Specifically, after treatment with MAPs, skin samples exhibited significantly lower melanin indices and surface differences in pigmentometer and confocal microscopy examinations, indicating its promising efficacy in inhibiting hyperpigmentation (Liu et al., 2020).

### 3.2 Powerful antibacterial effects

In addition to its anti-inflammatory and anti-pigmentation effects, MAPs also exhibit antibacterial activity. Research shows that MAPs, by binding with functional peptides (MAP-FPs), not only inhibit the growth of Gram-negative bacteria but also rapidly kill the bacteria. In *in vitro* experiments, MAP-FP peptides can kill various Gram-negative bacteria, such as *Escherichia coli* and *Salmonella*, within just 10 min. More importantly, these MAP-FP peptides maintain good stability and low cytotoxicity under high-temperature conditions, demonstrating their high safety for practical applications (Kim DY. et al., 2024).

The antibacterial properties of MAPs also provide new directions for their development as wound dressing materials. With their strong adhesiveness and antibacterial activity, MAPs can help wounds heal quickly and prevent bacterial infections, especially showing great potential in controlling drug-resistant bacterial strains. This offers a novel biomaterial option for wound healing and skin treatment (Fichman et al., 2021).

MAPs have also demonstrated performance in antibacterial coatings for orthopedic implants. They can firmly bind to the surfaces of implants, such as titanium alloys, and provide controlled drug release capabilities. This coating uses pH-dependent metal coordination to release antibiotics in the acidic environment caused by infection. Specifically, the antibacterial coating of MAPs can quickly release antibiotics in response to changes in bacterial concentration, showing excellent antibacterial effects, especially in early and late infections after implantation (Choi et al., 2024). This feature is of great clinical significance in preventing and treating complications of orthopedic implants, particularly prosthetic failure caused by bacterial infections.

### 3.3 MAPs in tumor immunotherapy

The application of MAPs in tumor immunotherapy has also made significant progress. In cancer treatment, immune checkpoint blockade (ICB) therapy has shown great potential; however, current ICB therapies are often accompanied by serious systemic autoimmune reactions and other side effects. To address this issue, Joo KI et al. developed an immune bio-glue (imuGlue) local drug delivery platform based on MAPs. This platform utilizes the underwater adhesion properties of MAPs to retain anti-PD-L1 drugs at the tumor site and enable on-demand drug release in response to changes in the tumor microenvironment. Studies have shown that imuGlue significantly enhances antitumor effects in triple-negative breast cancer and melanoma models while reducing systemic toxicity (Joo et al., 2020). This local release mechanism effectively prevents the rapid diffusion of anti-PD-L1 drugs in the body, alleviating their systemic toxicity, thus providing a new therapeutic strategy for cancer immunotherapy.

Additionally, MAPs have been applied in developing new tumor immunotherapy strategies, particularly in overcoming the immunosuppressive tumor microenvironment. By combining immune enhancers and photosensitizers with the MAP carrier, precise drug co-delivery has been achieved, which not only promotes tumor photothermal ablation but also expands the variety of tumor antigens. This enhances the persistence and effectiveness of local immunotherapy (He et al., 2025). This MAP-based drug delivery system can modulate the tumor microenvironment, overcome immune suppression, and enhance antitumor immune responses, further advancing the research into combination immunotherapy.

**FIGURE 2:**
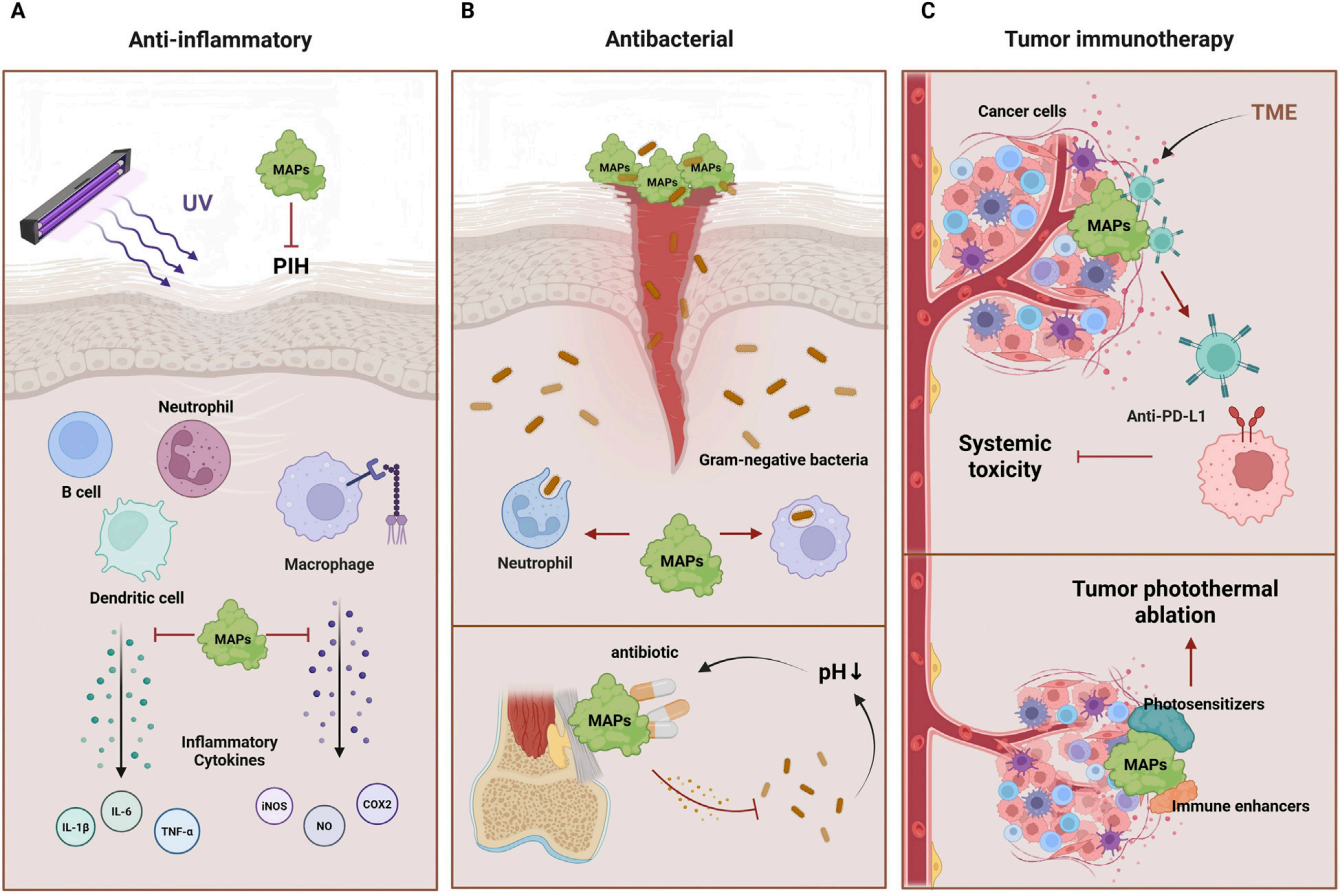
The pharmacological effects of MAPs. **(A)** MAPs can inhibit the occurrence of skin inflammation and PIH induced by LPS and UV radiation by suppressing inflammatory cytokines. **(B)** MAPs not only exhibit activity in inhibiting the growth of Gram-negative bacteria but can also release antibiotics in the acidic environment induced by infections, preventing bacterial infections. **(C)** MAPs can retain anti-PD-L1 drugs at the tumor site and release the drugs on demand in response to changes in TME. Furthermore, by combining immune enhancers and photosensitizers into MAP carrier, tumor photothermal ablation is promoted, enhancing the efficacy of immunotherapy.

Figure 2 shows the pharmacological effects of MAPs in anti-inflammatory, antibacterial, and tumor immunotherapy.

## 4 Applications of MAPs in the biomedical field

Native mussel adhesive proteins (MAPs), particularly Mfp-1, Mfp-3, and Mfp-5, have been extensively studied as natural templates for bioadhesive development. These proteins play distinct roles in the mussel byssus and have also been directly employed in biology and materials fields.

Mfp-1, a high-molecular-weight protein rich in DOPA and lysine residues (Lin et al., 2007), is primarily responsible for forming protective and corrosion-resistant films on metal and oxide surfaces. Studies have demonstrated that Mfp-1 can self-assemble into stable, oxidation-resistant coatings that improve surface hydrophilicity and biocompatibility, suggesting potential use in biomedical implants and antifouling coatings (Baskaran et al., 2025).

Native Mfp-3 coatings have been shown to enhance cell attachment and spreading on polymer and glass surfaces, while Mfp-5 displays superior adhesion strength under seawater-mimicking conditions. These native MAP-based coatings have also been applied to modify tissue scaffolds, promoting osteoblast and fibroblast adhesion at cell–material interfaces. Proteins with highest levels of DOPA, such as Mefp-3 (20 mol.%) and Mefp-5 (30 mol.%), exhibit wet adhesion to both inorganic and organic substrates through multiple catechol-mediated interactions. By integrating Mfp-3 and Mfp-5 peptide motifs into elastin-like polypeptides, adhesive hydrogels with tunable mechanical properties, adhesion, and excellent cytocompatibility were developed, providing a novel strategy for designing bioinspired adhesive materials for biomedical applications (Park et al., 2019; Li Z. et al., 2025).

Also MAPs have been applied as surface coatings for implants to enhance osseointegration, as adjuncts in guided bone regeneration to improve osteoinductivity (Kwan et al., 2023). Their unique adhesive properties and biocompatibility make them crucial for wound healing, skin repair, and trauma care (Santonocito et al., 2019).

### 4.1 Recombinant mussel adhesive proteins

Currently, the acquisition of MAP mainly involves directly extracting the natural adhesive protein components from the foot glands of mussels. The natural sources are rather limited, the production process is complex, the cost is high, and it is prone to solidification, which greatly restricts the application of mussel adhesive protein. To break through this production bottleneck, recombinant mussel adhesive proteins (recombinant Mussel Adhesive Proteins, rMAPs) have emerged. rMAPs are proteins expressed and produced in microbial hosts such as *Escherichia coli* through genetic engineering, and they have similar amino acid sequences and functional characteristics to natural MAPs (Kaushik et al., 2015). The similarities and differences between natural and recombinant mussel adhesive proteins are shown in Table 1.

**TABLE 1:** A comparison of the similarities and differences between natural and recombinant mussel adhesive proteins.

Project	Natural mussel adhesive proteins (MAPs)	Recombinant mussel adhesive proteins (rMAPs)
Source	Extracted from *Mytilus edulis* (natural source)	Synthesized through genetic engineering in host cells (*e.g., E. coli*, yeast)
Structure	Composed of various amino acids, rich in 3,4-dihydroxyphenylalanine (DOPA) residues, with complex structural domains, often including adhesive and cross-linking domains	Engineered via genetic synthesis to resemble natural MAPs but can be optimized or modified for specific functions
Molecular Properties	Contains natural DOPA residues, which confer strong underwater adhesion ability, along with good biocompatibility and biodegradability	Engineered with controlled properties, such as antioxidant, anti-inflammatory, antibacterial effects; may require chemical modifications to enhance functionality
Advantages	1. Natural biocompatibility and biodegradability 2. Strong underwater adhesion (The adhesion of Mfp-1 to mica is 1 mJ m^-2^, while that of Mfp-3 is 6 mJ m^-2^) (Waite, 2017) 3. Widely used in wound healing and tissue repair	1. Customizable through genetic engineering 2. Higher yield and reproducibility 3. Enhanced pharmacological effects
Disadvantages	1. Complex and expensive extraction process 2. Low yield 3. Underwater adhesion efficiency may be influenced by environmental factors	1. Potential toxicity in expression systems 2. Challenges in correct protein folding and functionality 3. Potentially high production costs
Applications	Primarily used in biomedical fields such as tissue engineering, wound healing, and skin repair	Suitable for biomedical applications, drug delivery systems, tissue regeneration, cosmetics, and precision medicine
Development Trends	Requires improvement in extraction technologies to enhance production efficiency	Ongoing advancements in genetic engineering and protein design make rMAPs a promising field, especially in biomedical and environmental applications

Despite this, the development of rMAPs still faces a core challenge: how to efficiently and accurately introduce its key active groups - DOP - into the recombinant protein. DOPA is the chemical basis that endows mussel adhesion protein with strong wet adhesion ability. Currently, the recombinant mussel adhesion protein produced through the *Escherichia coli* expression system requires tyrosinase modification to convert tyrosine residues into 3,4-dihydroxyphenylalanine (DOPA), but it still faces low DOPA conversion rate and low modification efficiency, making it difficult to obtain the adhesion properties similar to those of the naturally extracted MAP. To overcome this problem, researchers have developed various optimization strategies. Adjusting the synthesis pathways or co-expressing tyrosyl-tRNA synthetase (TyrRS), which recognizes DOPA, can significantly improve the incorporation rate of DOPA, thereby enhancing its adhesive properties (Jeong et al., 2020). Moreover, the catalytic action of tyrosinase can directly convert tyrosine into DOPA without additional cofactors. Researchers have constructed functionalized tyrosinase to further improve the modification efficiency of DOPA and developed tyrosinases that remain stable under neutral and alkaline conditions. The application of these enzymes not only increases the yield of rMAPs but also enhances their potential in skin repair and tissue engineering (Kim S. et al., 2021).

Beyond traditional genetic engineering methods, another innovative direction involves synthesizing polymers that mimic the structure and function of MAPs. Studies have shown that by polymerizing monomers containing repetitive peptide segments via ring-opening polymerization reactions, synthetic polymers resembling the structure of natural MAPs can be prepared. These polymers exhibit superior adhesive performance compared to natural MAPs on certain surfaces, and this strategy holds potential for various biomedical applications, such as the fixation of living cells (Berger et al., 2022).

An important research direction for rMAPs is the development of composite co-precipitates based on MAPs. By combining rMAPs with charged macromolecules to form complex co-precipitates, these composite materials demonstrate excellent adhesive properties in underwater environments. These materials have potential applications in biomedical and tissue engineering, particularly as a new bio-adhesion solution for non-invasive surgical repairs (Kim HJ. et al., 2017).

Compared to natural MAPs, rMAPs offer significant advantages in terms of production cost and application scope. Mfp-151, obtained through recombinant DNA technology by tandem expression of Mfp-1 and Mfp-5 proteins found in mussels, represent a typical structure of natural MAPs (Yu et al., 2013; Lu et al., 2013). The rMAPs obtained through this method possess structural characteristics similar to those of extracted MAPs, including DOPA and lysine residues. These structural features grant rMAPs excellent adhesive properties and biocompatibility. The DOPA group enhances the adhesive performance of the product, while the lysine group, with its positive charge and susceptibility to oxidation, promotes cell migration and wound healing. Mfp-151, through its unique physical properties, can not only form a protective biofilm over wounds but also create a microscopic discontinuous layer on the wound surface, facilitating tissue repair and protection.

With the continuous progress in production technologies, including improving DOPA incorporation rates, enhancing tyrosinase catalytic efficiency, and developing synthetic polymers, rMAPs will exhibit broad application prospects in the biomedical field as materials with excellent biocompatibility and adhesive properties. This makes them a critical research direction in biomedical materials (Horsch et al., 2018; Xue et al., 2025a).

Although rMAPs exhibit remarkable adhesive and biological characteristics, it is also important to distinguish their advantages from those of purely catechol-based synthetic systems. Synthetic materials such as polydopamine (PDA) coatings and catechol-grafted polymers share the same DOPA/catechol chemistry and have been widely applied as versatile, cost-effective, and scalable surface modifiers (Omidian and Wilson, 2024).

The primary strength of synthetic catechol systems lies in their superior practicality and scalability. Polydopamine’s “dip-coating” process is extremely straightforward (Saraf et al., 2024), while catechol-grafted polymers allow for tunable mechanical properties and compatibility with standard industrial processes, making them more competitive for large-scale, cost-sensitive applications. In contrast, rMAPs offer molecular precision and programmability. Their key advantage lies in the ability to integrate specific functional domains via genetic engineering, enabling more specific and tougher adhesion (Xue et al., 2025a), enhancing anti-inflammatory and antimicrobial properties (Kim S. et al., 2024) and so on, with greater potential in fields demanding high biocompatibility, such as biomedicine.

Therefore, the choice depends on the application scenario. rMAPs offers unique solutions for high-value precision biomedical applications, while the synthetic catechol system is more practical and feasible in general fields that require large-scale and economical surface treatment.

### 4.2 Wound healing and tissue repair

rMAPs exhibit adhesive properties, anti-inflammatory, antibacterial, antioxidant effects, and promotes cell adhesion, which can accelerate the wound healing process and is widely used in the development of various wound healing materials (Dai et al., 2024). For example, studies have shown that nanofiber scaffolds made by mixing rMAPs with polycaprolactone (PCL) demonstrate accelerated regeneration in a rat skin wound healing model. These nanofiber scaffolds not only provide good mechanical strength and a cell-friendly growth environment but also effectively adsorb growth factors, promote the growth of keratinocytes, and thus enhance wound healing (Kim BJ. et al., 2017).

In addition to nanofiber scaffolds, rMAPs are also applied in other types of biorepair materials. Researchers have developed self-healing materials using the adhesive and biocompatible properties of rMAPs, such as a polyurethane-urea self-healing material based on rMAPs and aromatic disulfide bonds. This material can efficiently self-heal at room temperature and possesses strong water resistance and excellent mechanical properties, demonstrating broad potential for various biomedical applications (Liu et al., 2021). This material is particularly suitable for making wound dressings and trauma repair products, effectively working in moist environments.

rMAPs can also accelerate wound healing, especially when treating skin infections and burns. When combined with other natural materials like guar gum (CG), rMAPs can be used to create hydrogels with self-healing, antibacterial, anti-inflammatory, and cell-proliferating properties. These composite hydrogels not only possess excellent mechanical strength and biocompatibility but also effectively promote wound healing and reduce necrosis progression at burn sites, thereby avoiding severe local inflammatory reactions (Cui et al., 2020; Han et al., 2024).

Another important application of rMAPs is in wound hemostasis and bioadhesive fields. Traditional hemostatic agents often suffer from insufficient adhesion and instability, while nanofiber dressings based on rMAPs can form stable physical barriers at the wound site, effectively promoting platelet aggregation and blood clotting (Wang Z. et al., 2024). For example, a double-sided nanofiber dressing composed of rMAPs and silk fibroin (SF) can promote hemostasis through the inner layer of rMAPs, while the outer SF layer provides physical protection, reducing the risk of wound infection. This dressing not only possesses biodegradability but also provides long-term protection in the body without causing a long-term burden on tissues (Lee et al., 2024).

To better understand the unique advantages of MAPs in comparison to other commonly used bioadhesives, we have summarized key properties such as wound closure rates, infection control, cytokine profiles, and adhesion metrics. Table 2 presents a comparative analysis of MAPs and traditional bioadhesives like collagen, gelatin, and silk fibroin, highlighting the distinctive benefits of MAPs, particularly their superior performance in moist environments and their ability to modulate the immune response. This comparison emphasizes the multifaceted pharmacological effects of MAPs, which arise from the synergistic interplay of multiple motifs rather than a single residue.

**TABLE 2:** A comparison of MAPs and other natural biomaterials.

Project	MAPs	Gelatin	Collagen	Silk fibroin	Keratin
Source	Natural secretions from mussel foot glands; recombinant MAPs and synthetic catechol analogs	Derived from collagen	Animal tissues	Silkworms	Human hair, wool, and feathers
Key functional motifs	DOPA (catechol), Lys, Gly repeats	Consists of N-acetyl-d-glucosamine (acetylated unit) and β-(1-4)-linked d-glucosamine (deacetylated unit) dispersed at randomly	Triple helix structure composed of three polypeptides of amino acids	β-sheet crystals (Ala–Gly repeats)	A stable three-dimensional network structure consist of hydrogen, ionic, ester, and Cys-rich disulfide bonds (Hiruthyaswamy and Deepankumar, 2025)
Wound closure/healing outcomes	85%–95% closure in 14–21 days (mouse/burn)	-	-	-	-
Infection control or cytokine modulation	Kill various Gram-negative bacteria; TNF-β↓ (Wu et al., 2024),	Neutral to mildly inflammatory	Mild immune response; no intrinsic antibacterial effect	Slight anti-inflammatory effect	Inhibit the adhesion of *E. coli* and *S. aureus*, preventing bacteria colonization and biofilm formation
Typical biomedical applications	Wound dressings, tissue adhesives (Khodve et al., 2025), bioactive coatings, hemostatic sealants, injectable hydrogels	Drug delivery carriers, temporary hydrogels, bioprinting inks	Dermal scaffolds, cartilage/bone repair membranes, cosmetic fillers	Sutures, film dressings, vascular grafts, scaffolds for skin and nerve regeneration	Hair/skin repair gels, fibroblast scaffolds, cosmetic applications

In conclusion, rMAPs have demonstrated unique biomedical value in wound healing and tissue repair applications. Their excellent biological adhesion, biocompatibility and biodegradability make rMAPs an ideal material for wound repair, as they effectively promote wound healing, haemostasis and antibacterial effects, as well as tissue repair.

### 4.3 Skin repair and treatment

rMAPs have anti-inflammatory properties that help alleviate skin itching and other discomforts, showing significant potential in the treatment of various skin diseases. For example, in scar repair after deep skin wounds, abnormal collagen fiber remodeling is one of the key factors that lead to poor healing. rMAPs, by forming a collagen-targeting adhesive with specific glycosaminoglycans (GAGs), can specifically bind to type I collagen and regulate the rate and extent of collagen fiber generation, effectively promoting wound regeneration and collagen synthesis (Jeon et al., 2017). Additionally, this collagen-targeting adhesive improves the structure and function of skin tissue by restoring normal collagen fiber architecture, exhibiting a good scar inhibition effect.

Moreover, rMAPs have shown unique therapeutic effects in treating inflammatory skin diseases such as facial erythema and rosacea. Due to the complex etiology of rosacea, existing treatments often have limited efficacy. However, rMAPs, delivered via microneedles, can improve the skin’s immune response, reduce inflammatory infiltration, and restore abnormal neurovascular regulation. In a clinical trial, after microneedle treatment with rMAPs, patients showed significant relief from erythema, flushing, and capillary dilation symptoms, with no noticeable adverse reactions during the treatment (Luo et al., 2024). Similarly, rMAPs have also shown positive clinical effects in treating sensitive skin (SS). When delivered via microneedles, rMAPs improve symptoms such as dryness, tightness, and flaking. During treatment, patients’ skin reactions were effectively alleviated, with no significant side effects or relapse, demonstrating good safety and high patient satisfaction (Dong et al., 2023).

With continued research, more mature products based on rMAPs have successfully been launched and applied in clinical practice. Currently, there are various skin repair products on the market that use rMAPs as a base, such as rMAP wound healing dressings and rMAP cream dressings. These products have been clinically validated, especially in treating non-chronic wounds, where rMAPs have played a significant role. For instance, Zhanyan recombinant mussel adhesive protein, a rMAP-based product, is suitable for treating skin wounds post-laser, photorejuvenation, dermatitis, eczema, sensitive skin, acne, etc., helping repair and protect these superficial wounds.

Laser and photorejuvenation technologies are commonly used to treat pigmentary disorders, skin aging, and scars, and the superficial wounds formed during treatment typically require rapid repair. rMAPs can provide protection for these wounds, promote epidermal regeneration, and reduce postoperative discomfort (Fu et al., 2019). Chemical peeling, which causes superficial skin damage through substances like fruit acids, can also be alleviated by rMAPs, which helps reduce redness, swelling, and accelerate recovery (Lee et al., 2019).

Overall, the role of rMAPs in skin repair and treatment has been extensively researched and applied. The launch of mature products marks the transition of MAP from laboratory research to clinical practice, with broad market prospects.

### 4.4 Bone repair and regeneration

The role of rMAPs in bone repair and regeneration has gradually gained attention, especially in promoting bone tissue regeneration and improving the stability of medical implants (Yang K. et al., 2024). MAP coatings can support the attachment and proliferation of human keratinocytes and chondrocytes, indicating that MAPs provide a good growth environment for cells during the bone repair process (Ivarsson et al., 2021).

In recent years, researchers have used bioengineering methods to combine MAPs with other bioactive molecules, successfully creating various surface coatings and scaffolding materials with bone-promoting functions. For example, when MAPs are mixed with gelatin and loaded onto the surface of nano-titanium tubes, they can promote osteogenic differentiation and bone formation by activating the FAK-PI3K-MAPKs-Wnt/β-catenin signaling pathway (Kim JE. et al., 2021). MAPs play a role in bone repair by promoting the adhesion of osteoblasts and activating FAK, thereby regulating the PI3K/Akt signaling pathway. This not only enhances the proliferation and survival of osteoblasts, but also stabilizes β-catenin and promotes its nuclear translocation. Together with the Wnt signaling pathway, they form a synergistic effect, jointly promoting osteoblast differentiation and extracellular matrix remodeling, thereby accelerating the bone integration process around the implant. This pharmacological mechanism has been fully verified in in vitro cell experiments and *in vivo* rat model experiments in the literature, providing a scientific basis for the application of MAPs in the surface modification of dental implants. By combining MAPs with silica nanoparticles (SiNPs) through the fusion of R5 peptides, a micro-rough structure can be formed on the surface of titanium alloy implants, effectively promoting the proliferation and differentiation of pre-osteoblasts, improving implant stability and long-term effectiveness (Jo et al., 2017).

Furthermore, when MAPs are combined with hyaluronic acid (HA), the resulting co-precipitates significantly enhance the blood resistance and mechanical properties of bone graft materials, thereby promoting bone regeneration. This material can effectively improve osteoconductivity and accelerate the regeneration of bone tissue while maintaining the stability of bone grafts (Kim et al., 2016).

In the application of guided bone regeneration (GBR), MAP-loaded collagen membranes also show good results. Experimental studies have found that MAPs effectively function as barrier membranes, supporting bone regeneration and promoting the formation of new bone. Compared to traditional cyanoacrylate (CA)-loaded collagen membranes, MAP-loaded collagen membranes demonstrate better biocompatibility and fewer inflammatory responses, indicating their promising application in bone repair (Song et al., 2018).

rMAPs can also be combined with multifunctional bioactive peptides to improve bone regeneration outcomes. Research shows that materials containing MAPs and multifunctional bioactive peptides can enhance cell aggregation and mineral deposition in the bone healing process by promoting macrophage migration (Lee et al., 2023).

MAPs are also used in the surface modification of titanium alloys to enhance their clinical application in dentistry. By combining with titanium nano-network structures (TNS), MAPs significantly improve cell attachment and proliferation and promote the expression of bone-related genes. In *in vivo* experiments, titanium alloys with MAP coatings promoted new bone growth at the bone-implant interface, further verifying their potential in bone regeneration (Yin et al., 2019).

Overall, rMAPs play a multifaceted role in bone repair and regeneration, promoting osteoblast function and improving implant biocompatibility and stability. By combining with other bioactive molecules and materials, MAPs provide innovative solutions for bone repair, particularly in the clinical applications of dental and orthopedic treatments (Jo et al., 2023; Yun et al., 2025).

### 4.5 Tissue engineering

The application of rMAPs in tissue engineering has been increasingly recognized, particularly in areas such as biological adhesion, regenerative medicine, and material surface functionalization. rMAPs can effectively promote the adhesion of biomaterials to tissues through their underwater adhesion properties, demonstrating outstanding performance in improving the biocompatibility and biological functionality of biomaterials (Fang et al., 2017).

In cardiovascular implants, MAPs can enhance cell proliferation and migration by fusing with growth factors (such as VEGF) and extracellular matrix proteins (such as fibronectin). Through this biofunctionalization approach, MAPs help improve cardiac remodeling and myocardial protection, particularly in the repair following myocardial infarction, significantly extending the retention time of therapeutic peptides and promoting stable adhesion between the therapeutic patch and the host myocardium through their natural strong adhesive properties (Lim et al., 2021). Therefore, MAPs are widely used in surface modification of vascular stents. By combining MAPs with other bioactive molecules (such as NO-generating compounds and endothelial progenitor cell-targeting peptides), they can effectively prevent thrombosis, smooth muscle cell proliferation, and promote the recruitment and proliferation of endothelial progenitor cells, thereby reducing the occurrence of restenosis in stents (Yang Z. et al., 2020). This research shows that MAPs not only play a role in biological adhesion but also improve the clinical outcomes of stents by regulating cellular responses (Wang et al., 2020).

Moreover, rMAPs have become ideal materials for wound healing (Yao et al., 2022). Traditional suturing and stapling methods may cause significant tissue damage and inflammatory responses, especially in the repair of internal organ wounds. MAPs offer a new approach to address these issues. For instance, by developing a bilayer microneedle patch based on MAPs, researchers have achieved superior wound sealing capability, effectively preventing leakage and fluid infiltration in internal organ wounds (Jeon et al., 2019). This innovative bioadhesive material not only exhibits excellent adhesion but also provides reliable repair effects in dynamic environments, making it suitable for both external and internal tissue wound healing.

Unlike traditional synthetic adhesives, rMAPs also perform exceptionally well in underwater adhesion. Copolymers based on MAPs, such as poly(catechol-styrene), exhibit underwater adhesion strengths that even surpass the natural adhesion ability of live mussels (North et al., 2017). This adhesion property is particularly significant in saline environments, offering new solutions for underwater wound repair and medical devices that need to function in moist or submerged environments. For example, MAP-based water-incompatible bioadhesives have been successfully used to close urethral and vesicovaginal fistulas, demonstrating excellent adhesion and durability in wet environments. This feature makes them a potential repair material for urological and other internal surgeries (Kim HJ. et al., 2015; Kim HJ. et al., 2021).

In ophthalmic surgeries, researchers have successfully achieved sutureless amniotic membrane transplantation using the FixLight protein bioadhesive developed from MAPs. This adhesive can rapidly solidify and has good biocompatibility with ocular tissues, greatly simplifying the surgical process and reducing postoperative complications (M et al., 2021).

Finally, rMAPs also show broad prospects in the repair of internal organs and the implantation of medical devices. By adjusting the physical properties and biodegradability of the adhesives, customized bioadhesive patches can provide strong underwater adhesion and controllable degradation properties to meet the specific needs of different organs, thereby playing a role in various biomedical applications (Yang et al., 2024b).

MAPs can be used to construct thermoresponsive tissue adhesive hydrogels to promote soft tissue regeneration. By combining MAPs with poly(N-isopropylacrylamide) (PNIPAM), an injectable hydrogel system was prepared that activates and enhances tissue adhesion and moisture retention at body temperature. Compared to the use of PNIPAM gel alone, MAP-PNIPAM hydrogels exhibit significantly stronger tissue adhesion and better hydration, thus improving the effectiveness of adipose tissue engineering, such as cell survival rates and integration with host tissues (Jeon et al., 2020).

In summary, the role of MAPs in tissue engineering is not limited to their underwater adhesion properties but also involves promoting cell growth and tissue repair through their interaction with biomolecules. They offer new insights into the functionalization of biomaterial surfaces, with wide applications in regenerative medicine, cardiovascular therapy, and tissue engineering (Hu et al., 2024).

### 4.6 Targeted drug delivery and therapeutic applications

Due to the ability of rMAPs to firmly bind to biological tissues through DOPA residues, their application in drug delivery systems has become a highly promising option. A drug delivery system based on MAPs can form a coordination complex with iron (III) by modifying the DOPA residues, enabling drug release in acidic environments and demonstrating excellent pH responsiveness. This property gives it a significant advantage in anticancer drug delivery, especially in local and targeted therapies. Drug-loaded polymeric nanoparticles modified with MAPs can effectively enter cancer cells via the cell membrane and release drugs in the low pH microenvironment, enhancing local drug accumulation and reducing systemic toxicity (Kim BJ. et al., 2015).

The excellent adhesion and biocompatibility of rMAPs make them an ideal choice for the preparation of functionalized nanoparticles. MAPs can react with dopamine to form polydopamine (PDA) nanoparticles, which exhibit good near-infrared absorption and photothermal effects, making them suitable for use as a targeted drug delivery platform in photothermal therapy for cancer (Zhang et al., 2018; Chen et al., 2020). Mussel adhesive protein-modified polyacrylic acid nanoparticles can form micro-needle patches with strong adhesive properties, offering excellent biocompatibility and enabling continuous drug release and long-term targeted therapeutic effects (Yang et al., 2024c). These nanoparticles effectively inhibit tumor growth, and after a single administration, the drug remains at therapeutic concentrations in the tumor site (Yang et al., 2025; Jeong et al., 2025). This sustained release effect makes MAPs a highly promising tool in cancer therapy.

A spray-based drug delivery system utilizing MAPs has also been proposed as an innovative cancer treatment strategy (Feng et al., 2024). By spraying MAP nanoparticles loaded with chemotherapy drugs directly onto the tumor surface, the drug residence time on the tumor surface can be increased, effectively inhibiting tumor growth and significantly improving treatment efficacy while reducing adverse reactions (Jeong et al., 2018).

Furthermore, MAPs have made significant progress in nerve regeneration applications. By combining MAPs with extracellular matrix (ECM) peptides, electrospun nanofiber conduits were fabricated, which not only enhanced the adhesion and proliferation of nerve cells but also promoted neuronal regeneration through contact guidance. This multidimensional strategy incorporating MAPs provides a new direction for nerve injury repair, particularly in nerve grafting and nerve conduit design, with great potential for application (Cheong et al., 2019).

rMAPs also play an important role in stem cell delivery after myocardial infarction. Drug carrier systems APICLS prepared using MAPs not only improve stem cell encapsulation efficiency and survival rates but also promote the integration of cells with damaged myocardial tissue, enhancing cell persistence and paracrine effects, thereby aiding in heart repair and regeneration (Park et al., 2020).

The applications of rMAPs demonstrate their diversity and powerful functionality in drug delivery and targeted therapy. Through modification and combination with other substances, rMAPs can achieve more precise and sustained drug release, enhance drug efficacy, and reduce systemic toxicity (Xu Z. et al., 2024). Whether in microneedle systems for cancer treatment or in nerve repair applications, MAPs, with their unique adhesive properties and biocompatibility, offer multiple potential therapeutic approaches and drive innovation in biomedical materials (Liu et al., 2024).

### 4.7 Other applications

rMAPs have shown significant effects in promoting angiogenesis, especially in enhancing endothelial cell repair. After vascular injury, repair work is often challenging because the original vascular structure cannot be fully restored. In recent years, therapeutic angiogenesis has been considered a promising strategy; however, there is a lack of effective methods to simulate the *in vivo* angiogenesis process. To address this, Park TY et al. developed a novel therapeutic angiogenesis platform based on MAPs. Through their high adhesion and strong stickiness at lesion sites, rMAPs can release angiogenesis-promoting growth factors in a controlled, targeted manner, thus facilitating vascular repair. By working synergistically with platelet-derived growth factor (PDGF) and vascular endothelial growth factor (VEGF), this platform successfully achieved effective angiogenesis *in vivo*, demonstrating its great potential for treating ischemic diseases (Park et al., 2021).

Endothelial cells, as the primary constituents of blood vessels, are crucial for vascular repair. Research has shown that complexes formed by MAPs and exogenous VE-cadherin extracellular domains can effectively promote the adhesion of endothelial cells and endothelial progenitor cells, accelerating the vascular repair process. Specifically, this complex significantly increased the adhesion of endothelial cells and progenitor cells *in vitro* and, through interaction with VE-cadherin, promoted the aggregation and functional recovery of endothelial cells (Yang D. et al., 2018).

In clinical applications, vascular stents often need to accelerate the endothelialization process to reduce the risk of thrombosis. Studies have shown that MAP-coated stents quickly attract endothelial progenitor cells after implantation, forming a smooth endothelial layer within a short period. This reduces platelet adhesion and the formation of neointima, providing strong support for the biocompatibility and long-term efficacy of vascular stents (Yang D. et al., 2020).

rMAPs are also widely used in surface modification of biodegradable materials. For example, a composite film formed by MAPs and chitosan can effectively control the degradation rate of magnesium alloys in body fluids. This composite film not only enhances the corrosion resistance of magnesium alloys but also significantly improves their biocompatibility, providing a safer guarantee for the use of medical magnesium alloys (Jiang et al., 2016).

Moreover, MAPs have shown important applications in diabetes management. In the development of continuous glucose monitoring systems, researchers utilized the biocompatibility of MAPs to firmly immobilize glucose oxidase onto microneedle electrodes via electrochemical oxidation methods, achieving highly durable and selective glucose monitoring. Experimental verification showed that this MAP-based monitoring system exhibited performance comparable to commercially available systems, with high clinical application potential (Kim et al., 2019).

Finally, the anti-thrombotic properties of MAPs have led to their widespread use in blood compatibility modifications. Studies have found that MAPs can effectively inhibit platelet adhesion and activation, thus reducing the risk of blood clotting. By forming complexes with polysaccharides, MAPs not only enhanced their anti-platelet adhesion effects but also improved the biocompatibility of blood-contacting materials by reducing plasma protein adsorption (da Câmara et al., 2020).

**FIGURE 3:**
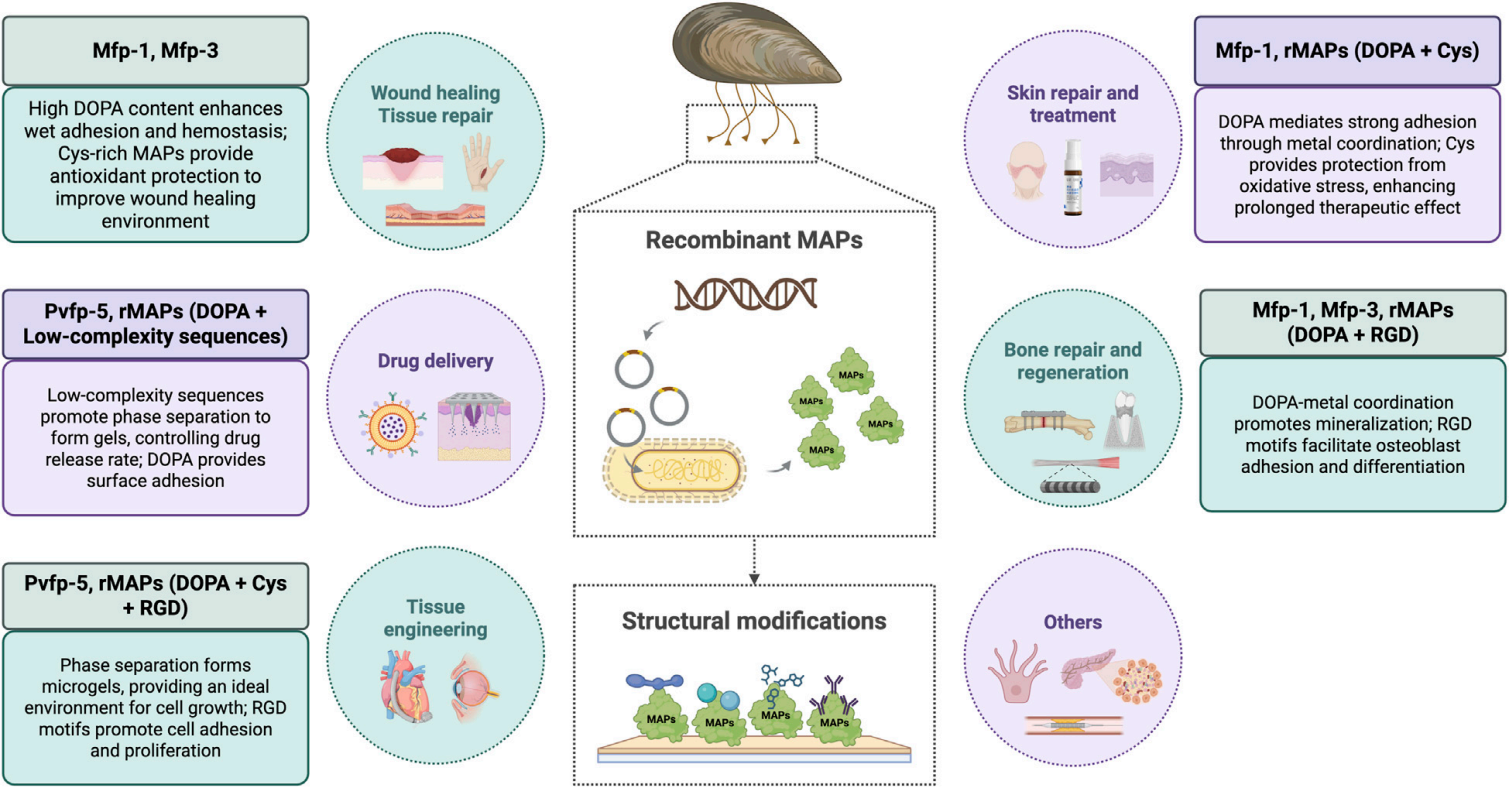
Recombinant MAPs and their applications in the biomedical field.

In summary, rMAPs have shown extensive application potential in vascular repair, tissue regeneration, medical device modification, and more (Figure 3). As research progresses, the potential of MAPs as a biomaterial will be further explored, providing more innovative therapeutic solutions for modern medicine.

## 5 Challenges and development directions of rMAPs

MAPs are considered ideal underwater adhesives, but they face several challenges, including recombinant protein production, toxicity control, and underwater adhesion efficiency. However, with the continuous development of new strategies, such as genetic engineering, light-activated technologies, and hybrid protein design, the application prospects of MAPs are becoming broader, providing significant technological support and innovation opportunities for biomedical repair, underwater engineering, and wound healing.

### 5.1 Current challenges facing rMAPs

MAPs have great potential as biomaterials due to their excellent adhesive properties. However, their application still faces many challenges, especially in terms of high-efficiency expression, functionalization, and use in wet environments.

Firstly, the natural yield of MAPs is relatively low, which limits their widespread use in practical applications. Although recombinant technology can increase production efficiency, due to the complexity of their expression systems and purification processes, particularly during large-scale production, balancing yield and cost remains a challenge. To address this issue, research has proposed improvement strategies through structural analysis. By altering the position and quantity of tyrosine, mutant proteins with enhanced expression levels, adhesive ability, and solubility have been successfully constructed (Xue et al., 2025b). Meanwhile, the CRISPR/Cas gene editing technology has been applied to optimize microbial expression systems, aiming to increase the expression level and solubility of recombinant proteins, thereby enhancing production. For instance, the CRISPR/Cas system was employed in engineered *Escherichia coli*, successfully improving the expression level and solubility of the target protein (Feser et al., 2023).

Secondly, although recombinant technology can provide a large amount of adhesive proteins, the resulting rMAPs often lack the complex three-dimensional structure and functional characteristics possessed by natural MAPs, leading to lower adhesive strength and stability. Some studies have enhanced adhesion performance using nanoparticle-based adhesives. Experiments have shown that the addition of metal ions can increase the cohesion between MAPs, thereby improving their adhesion (Baskaran et al., 2023).

Furthermore, the performance of rMAPs under different environmental conditions (such as varying pH, temperature, and humidity) may not be as stable as that of natural MAPs, limiting their application in complex environments. Specifically, rapid adhesion in underwater and wet environments remains a significant challenge. Hauf et al. adopted a strategy based on genetic code expansion, designing efficient aminoacyl-tRNA synthetases (aaRSs) and introducing photoactivatable atypical amino acids (such as ortho-nitrobenzyl DOPA, ONB-DOPA) into rMAPs. This innovative strategy enabled the MAPs to equip multiple ONB-DOPA molecules at specific sites, which were activated by ultraviolet light, significantly enhancing their adhesion in wet environments, providing a new direction for the recombinant production of underwater adhesives (Hauf et al., 2017). Additionally, stabilizers such as trifluoroethanol (TFE) and urea can maintain the unfolded state of proteins and promote their aggregation, forming strong underwater adhesives. This method reveals a universal principle for converting ordinary proteins into strong underwater adhesives, showcasing the rich potential of proteins from biological resources (Liu et al., 2023). It can also be optimized through AI-assisted algorithms, which can predict the amino acid substitutions that can enhance the adhesion strength, stability and overall performance of rMAPs. AI helps identify the modifications that improve the functional characteristics of rMAPs, making them more stable and effective in biomedical environments (such as drug delivery and tissue engineering) (Jiang et al., 2025).

In addition to the material- and structure-oriented challenges of recombinant MAPs discussed above, it is also important to consider the limitations that arise when MAPs are explored as therapeutic agents themselves. Recombinant MAPs share the same chemical and structural complexity as natural MAPs, which may cause issues related to enzymatic degradation, oxidation, and limited systemic stability. Their large molecular size and high hydrophilicity make targeted delivery difficult, while their potential immunogenicity has not been fully evaluated. There is a lack of long-term safety and immunogenicity data. While rMAPs have good biocompatibility and biodegradability, systematic animal and clinical studies are still scarce, leaving uncertainties about chronic inflammatory responses and degradation products (Xue et al., 2025b). For example, the oxidation of phenolic compounds catalyzed by tyrosinase can generate bisphenols, quinones, and highly polymerized melanins, which may cause biocompatibility issues in some applications, particularly in biomedical fields (Axambayeva et al., 2018). Some applications of rMAPs during curing processes may release hydrogen peroxide, which could lead to localized cytotoxicity and affect immune responses around tissues. Therefore, when developing bioadhesives containing MAPs, it is essential to carefully monitor and control the generation of these byproducts to ensure biocompatibility for specific applications (Meng et al., 2017).

Meanwhile, when compared with clinically approved tissue adhesives, rMAPs show both advantages and shortcomings. Fibrin glue offers rapid hemostasis and biodegradability but limited mechanical strength. PEG-based hydrogels are tunable and biocompatible yet less adhesive under wet conditions. Cyanoacrylate adhesives provide strong adhesion and fast curing but cause local cytotoxicity and tissue irritation. In contrast, rMAPs balance bioadhesion and biocompatibility but currently lack the scalability and regulatory maturity for clinical adoption.

Moreover, large-scale production, batch variability, and regulatory classification as biologics may further complicate clinical translation. To overcome these hurdles, researchers and manufacturers can collaborate closely with regulatory bodies to establish clear guidelines for the approval of rMAPs. Early-stage clinical trials should prioritize evaluating the biocompatibility, toxicity, and long-term stability of rMAPs. This collaborative approach will help streamline the regulatory approval process and facilitate the clinical translation of rMAPs into therapeutic use (Jiang et al., 2025). Addressing these pharmacological and translational barriers will be crucial for realizing the full therapeutic potential of MAPs beyond their material-based applications.

### 5.2 Future research directions of rMAPs

The future research directions of rMAPs mainly focus on improving their production efficiency, functional modification, and expanding their applications across multiple fields. Researchers aim to optimize the production processes of rMAPs, improving expression systems and purification methods to reduce costs and increase yields. Simultaneously, functional modifications through genetic engineering will enhance their stability and adhesive strength in extreme environments.

Exploring the combination of rMAPs with nanomaterials, drug carriers, and other components to develop multifunctional composite materials for use in medical, environmental, and smart material fields is also a promising avenue. For example, researchers have developed a composite material based on MAPs and graphene oxide (GO), synthesized through a green aqueous solution. This composite material not only exhibits high tensile strength and elongation but also demonstrates extraordinary toughness, surpassing many existing composite materials (Kim et al., 2020). This green synthesis strategy avoids the use of organic solvents and surfactants, resulting in a lower environmental impact and highlighting the potential applications of MAPs in high-performance materials.

In precision medicine, the unique adhesive properties of rMAPs can be utilized to develop personalized biomaterials, such as drug delivery systems targeting specific lesions. Through genetic engineering and synthetic biology techniques, researchers can introduce specific functional groups into the structure of rMAPs, enabling them to target and treat tumors, infectious diseases, and other diseased tissues in precision medicine.

The future research of rMAPs not only holds significant medical and biotechnological applications but also provides inspiration for research on other materials. The unique properties of rMAPs, such as efficient underwater adhesion, good biocompatibility, and adjustability, offer valuable experience for developing new high-performance materials.

Currently, traditional surgical tissue adhesives fail to meet the ideal requirements for biological adhesives due to their insufficient adhesive strength in moist conditions and higher toxicity. To address this issue, researchers have developed a novel light-activated biological adhesive, LAMBA, based on mussel adhesion mechanisms and insect di-tyrosine crosslinking chemistry (Jeon et al., 2015). This adhesive exhibits much stronger adhesion on moist tissues than commercially available fibrin glues and has good biocompatibility. By regulating crosslinking reactions through light exposure, LAMBA can effectively close wounds on demand and promote wound healing, showing broad medical application potential, especially in seamless wound closure and visceral tissue repair.

To address fast adhesion in moist environments, catechol-based adhesives inspired by MAPs have attracted widespread attention (Ni et al., 2024). Studies have shown that catechol compounds with long-chain fatty groups can be used to prepare thin, optically transparent underwater adhesive strips. These adhesive strips are rapidly activated in water and possess strong adhesive strength. Moreover, Wei W et al. have successfully enhanced adhesion by designing synthetic peptides mimicking MAPs and utilizing their co-precipitation properties with water-repellent surfaces (Wei et al., 2016).

Although powerful underwater adhesives are highly attractive for biomedical and underwater repair applications, their success in practical use still faces challenges such as underwater curing, surface adhesion, and the balance between cohesion and adhesion forces (Kim E. et al., 2021). Researchers have designed a novel hybrid protein combining the amyloid protein’s zipper structure, spider silk’s flexible sequence, and the DOPA sequence of MAPs. This hybrid protein can self-assemble into semi-crystalline hydrogels, exhibiting extremely high strength and toughness, and shows underwater adhesion on various surfaces (such as plastics, tendons, and skin). Selective de-adhesion can be achieved through oxidation or iron chelation treatments. This study provides new ideas for the design and synthesis of multifunctional hybrid protein materials and shows potential for wide applications.

Finally, exploring the pharmacological effects and mechanisms of rMAPs is also a key direction for future research. In addition to traditional adhesive applications, rMAPs may also exhibit bioactivity, such as antibacterial, anti-inflammatory, or tissue repair-promoting effects. By deeply studying the mechanisms of rMAPs’ action and integrating cell biology and molecular biology techniques, their potential applications in wound healing, tissue engineering, and even immunotherapy could be uncovered. Therefore, future research should not only focus on the physicochemical properties of rMAPs but also emphasize the exploration of their biological functions and the mechanism analysis, unlocking more application scenarios.

In summary, rMAPs have broad application prospects in fields such as precision medicine and smart drug delivery. Genetic engineering and synthetic biology will provide strong technical support for their development, driving innovation in future medical, material science, and other applications (Lee et al., 2016).

## 6 Conclusion

MAPs, as a type of natural underwater adhesive molecules, exhibit tremendous application potential in various fields such as biomedicine, drug development, and material science due to their exceptional adhesive properties, biocompatibility, and environmental adaptability (Hollingshead et al., 2022; Sun et al., 2020). In particular, MAPs have become ideal carrier materials in drug delivery and tissue engineering due to their unique properties. With in-depth research into their pharmacological effects and mechanisms, the clinical application prospects of MAPs are increasingly broad, especially showing significant potential in precision medicine, wound repair, and biosensor fields (Aich et al., 2018).

The pharmacological effects of MAPs primarily lie in their excellent bio-adhesion ability and multifunctionality. Through the covalent bonding of amino acids such as tyrosine and lysine, MAPs form strong and stable adhesive forces, enabling them to remain attached stably in underwater environments (Burke et al., 2016). Meanwhile, MAPs exhibit low immunogenicity, allowing for long-term use *in vivo* without causing immune rejection, which contributes to their high biocompatibility. These characteristics provide MAPs with unique advantages in drug delivery systems. MAPs not only enhance the solubility and stability of drugs but also improve drug targeting, increase bioavailability, reduce side effects, and enable sustained and controlled drug release (Talebian et al., 2020). Therefore, MAPs have shown great potential in targeted drug delivery, cancer therapy, and vaccine carriers.

However, further research is needed to strengthen the safety assessment and long-term effects of MAPs in clinical applications. Although they exhibit good biocompatibility, their long-term biological safety in large-scale clinical applications still requires in-depth study (Jiao et al., 2024). Particularly in scenarios involving long-term implantation, such as tissue repair and wound healing, it is crucial to ensure that their degradation products are non-toxic and do not cause immune reactions (Yun et al., 2024).

Natural products, as valuable sources of pharmacologically active molecules, have long provided inspiration for modern drug discovery and biomaterial design (Sun et al., 2024; Han et al., 2025; Li X. et al., 2025; M’barek et al., 2025). The study of MAP exemplifies how nature’s molecular architectures can serve as blueprints for next-generation therapeutic and bioadhesive innovations.

In conclusion, rMAPs hold enormous application potential in drug development and biomedical fields. With technological advancements, particularly in genetic engineering and synthetic biology, rMAPs are expected to overcome current research bottlenecks and be applied in more clinical settings, such as personalized drug delivery systems, wound repair, and cancer treatment. Despite facing some challenges, with ongoing research, the multifunctionality and wide applicability of MAPs will make them an important material in biomedicine, driving the development of precision medicine and novel treatment methods.

## Glossary

aaRSs: Aminoacyl-tRNA synthetases
CMC: carboxymethyl cellulose
CA: cyanoacrylate
COX-2: cyclooxygenase-2
DA: dopamine
ECM: extracellular matrix
Fp1: foot protein 1
GAGs: glycosaminoglycans
GO: graphene oxide
CG: guar gum
GBR: guided bone regeneration
HRP: horseradish peroxidase
HA: hyaluronic acid
InaKC: ice-nucleation protein K
imuGlue: immune bio-glue
ICB: immune checkpoint blockade
iNOS: inducible nitric oxide synthase
LPS: lipopolysaccharide
MAPs: mussel adhesive proteins
PDGF: platelet-derived growth factor
PNIPAM: poly(N-isopropylacrylamide)
PCL: polycaprolactone
PDA: polydopamine
PIH: post-inflammatory hyperpigmentation
rMAPs: recombinant mussel adhesive proteins
SS: sensitive skin
SiNPs: silica nanoparticles
SF: silk fibroin
TNS: titanium nano-network structures
TFE: trifluoroethanol
TyrRS: tyrosyl-tRNA synthetase
UV: ultraviolet
VEGF: vascular endothelial growth factor
